# Rhegmatogenous retinal detachment repair—does age, sex, and lens status make a difference?

**DOI:** 10.1007/s00417-022-05674-x

**Published:** 2022-05-02

**Authors:** Viola Radeck, Horst Helbig, David Maerker, Maria-Andreea Gamulescu, Philipp Prahs, Teresa Barth

**Affiliations:** Department of Ophthalmology, University Hospital of Regensburg, Franz-Josef-Strauss-Allee 11, D-93042 Regensburg, Germany

**Keywords:** Age, Cataract surgery, Sex, Pseudophakia, Retinal detachment surgery, Vitrectomy

## Abstract

**Purpose:**

To analyze the correlation between lens status, age, and sex in the epidemiology and success rates of rhegmatogenous retinal detachment (RRD) surgery.

**Methods:**

The files of all consecutive patients undergoing vitreoretinal surgery for uncomplicated RRD between Jan 2005 und Dec 2020 were retrospectively reviewed. Successful outcome was defined as no retinal redetachment occurring within 3 months after surgery.

**Results:**

5502 eyes with uncomplicated primary RRD were included. Mean age of the patients was 61.1 years (± 13.6 SD). In the age group over 40 years, a male predominance was found. The percentage of pseudophakic RRD increased from 25 to 40% during the 15 years observation period. In the age group 50 to 69 years, patients with pseudophakic detachments were male in 786 out of 1079 cases (72.9%). In the same age group, 1285 of 2110 (60.9%) patients with phakic RRD were male. Overall, primary success rate after one procedure was 91.2% (5018 of 5502). In the phakic eyes, the primary success rate was higher in those eyes that underwent combined phacovitrectomy (93.0%), compared to those without simultaneous cataract surgery (88.7%; *p* = 0.002).

**Conclusion:**

The ratio of male and female patients with RRD varies between age groups. The proportion of pseudophakic RRD has increased within 15 years. The male predominance in RRD is stronger in pseudophakic than in phakic eyes. In phakic eyes with RRD, a combined phacovitrectomy yielded better anatomical results.

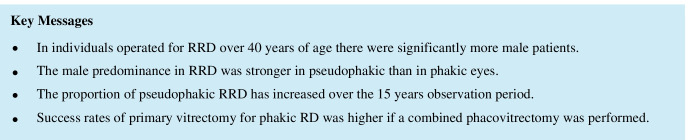



## Introduction

Rhegmatogenous retinal detachment (RRD) is a common surgical emergency in ophthalmology. Several factors contribute to an increasing occurrence of RRD, namely an increasing number of cataract surgeries and refractive lens exchanges, higher prevalence of myopia, and increasing life expectancy [1].

RRD is not a homogenous entity. It can affect all age groups, but with different clinical features. The most common presentation is an RRD caused by retinal tears associated with a posterior vitreous detachment (PVD). Other presentations include RRD with atrophic round holes in individuals without PVD and vitreoretinal degeneration/dystrophies. Most studies show a male predominance in RRD patients, and the reason for the observed sex ratio is however unclear.

Another factor that characterizes different subgroups of RRD is the lens status [2]. With the rising number of cataract operations, an increasing proportion of pseudophakic RRD can be expected. In phakic RRD on the other hand, vitrectomy is becoming increasingly common. Lens management during surgery is discussed controversially. Until today, there is no clear indication whether vitrectomy should be performed primarily without lens surgery or whether combined phacovitrectomy may be the better strategy.

In the present study, we have analyzed the impact of lens status, age, and sex on epidemiology and success rates of RRD surgery. We investigated whether RRD occurring in different age groups would also differ in other features such as sex distribution and success rates. We have also analyzed if the increasing number of cataract operations leads to a distinct increase of pseudophakic RRD during the duration of this study from 2005 to 2020 and how pseudophakia may affect the sex distribution of RRD. Finally, we have compared success rates of combined phacovitrectomy with those of vitrectomy alone.

## Materials and methods

The files of all patients undergoing primary RRD surgery between January 2005 and December 2020 in our department were reviewed. Included were all eyes with uncomplicated RRD undergoing buckle surgery or vitrectomy. Excluded were eyes with PVR grade C or more, eyes with a history of penetrating injury to the posterior segment of the eye, or a history of other vitreoretinal procedures in the past. Complicated cases such as malformations or retinopathy of prematurity (ROP) were also excluded from the analysis. High myopic eyes (> 6 Dpt) were included, but pathological myopia with RRD arising from macular holes in posterior staphylomata was excluded.

Failure was defined as diagnosis of redetachment documented in the patient file within 3 months after primary surgery. In eyes with silicone oil tamponade, the silicone was removed usually 3 to 6 months after primary surgery, which may lead to an underestimation of redetachments since those occurring after silicone removal were not included.

Our hospital is the only center for vitreoretinal emergencies in an area serving a population of about 2–3 million people. We assume that almost all redetachments were referred to us. In order to investigate, if we had been missing failures, in a subgroup of 1290 patients operated between 2007 and 2012, a more intense follow-up was conducted by contacting patients and their ophthalmologists. No additional redetachments were detected in addition to those already recorded in our own files, indicating that the overall number of overlooked redetachments would not be significant [3, 4].

The study was approved by the local ethic board (Ethikkommission der Universität Regensburg) and adheres to the principles of the declaration of Helsinki.

## Results

### Age distribution

**Fig. 1:**
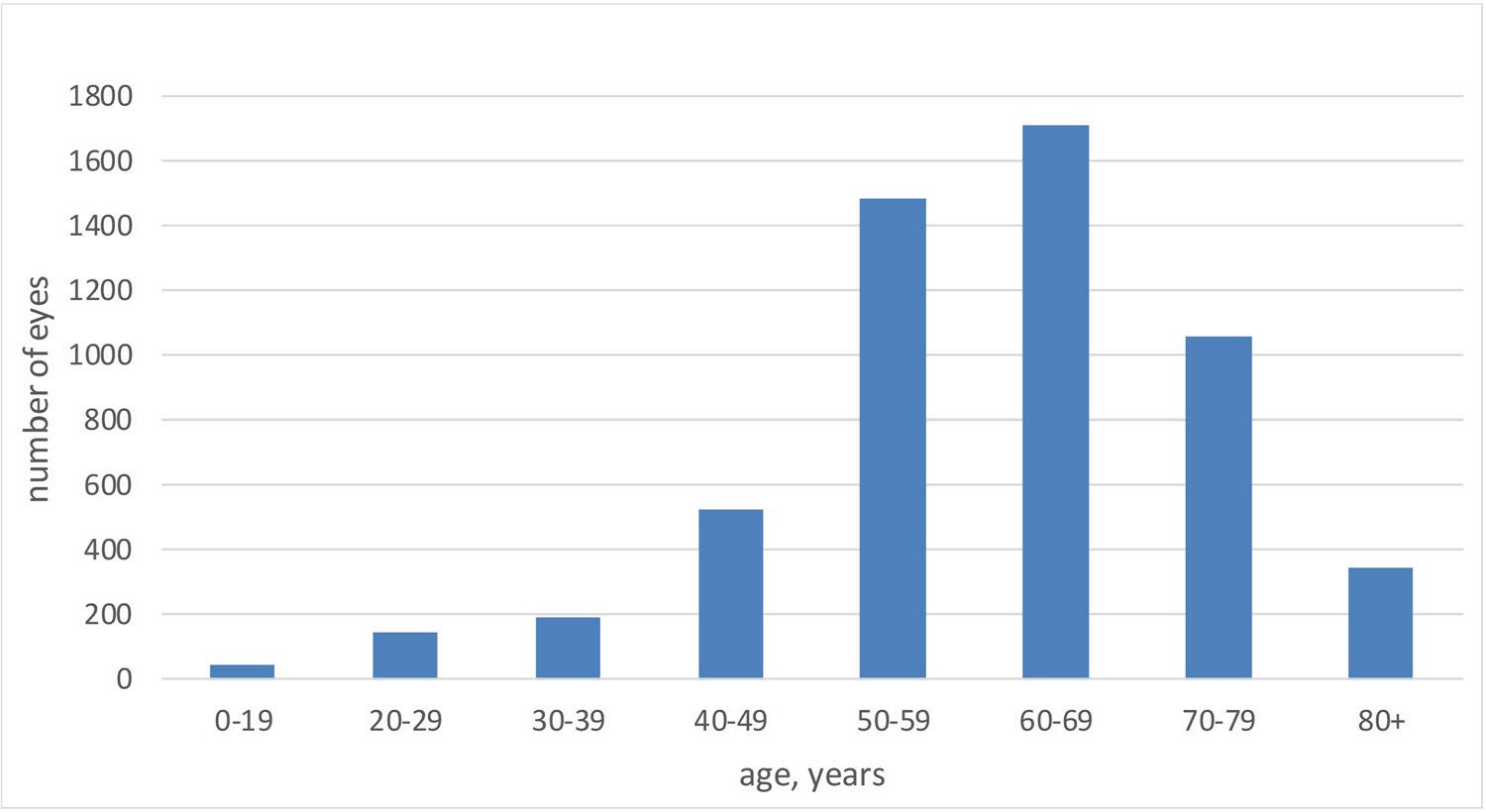
Number of eyes of patients with RRD in various age groups

Overall, 5502 eyes with uncomplicated primary RRD had VR surgery between January 2005 and December 2020 operated by 13 VR surgeons with different levels of surgical experience. Mean age of the patients was 61.1 years (± 13.6 SD). The age distribution is shown in Fig. 1. The youngest patient was 4, and the oldest was 98 years old.

### Procedures

A total of 810 (14.7%) eyes were treated with buckle surgery, 4692 (85.3%) with vitrectomy. In the vitrectomy cases, 95% received gas as a tamponade and 5% liquid silicone. The decision for buckle surgery or vitrectomy was left to the respective surgeon. In young patients with attached vitreous and atrophic holes or oral dialysis buckle surgery was preferred, most pseudophakic eyes were vitrectomized. Preferences however changed over the observation period, with an increasing proportion of eyes being treated with vitrectomy [3].

**Fig. 2:**
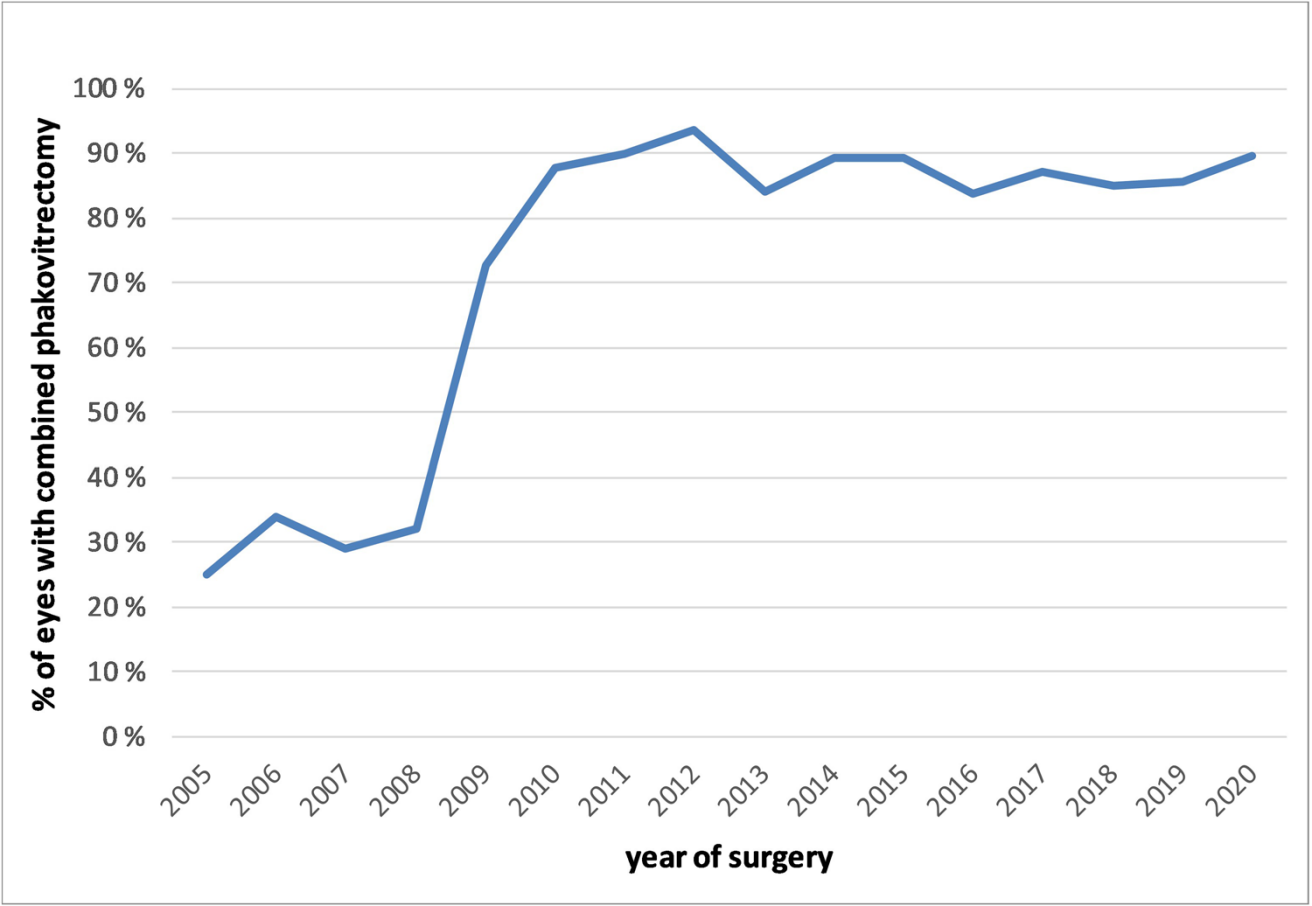
Proportion of phakic eyes with RRD treated with vitrectomy alone or combined phacovitrectomy

From the 2614 phakic eyes treated with vitrectomy, 2163 (82.7%) were operated combined with phacoemulsification and posterior chamber intraocular lens implantation. While in the early years only a minority of the phakic eyes was treated with combination surgery, at the end of the observation period, about 90% were operated as phakovitrectomy (Fig. 2). In 2008 to 2010, there was a paradigm shift to combining cataract surgery with vitrectomy. This was based on reports of good results with combined surgery, sufficient experience of the surgeons in both techniques, and the aim to avoid a second (cataract) surgery for the patient.

### Sex distribution in different age groups

**Fig. 3:**
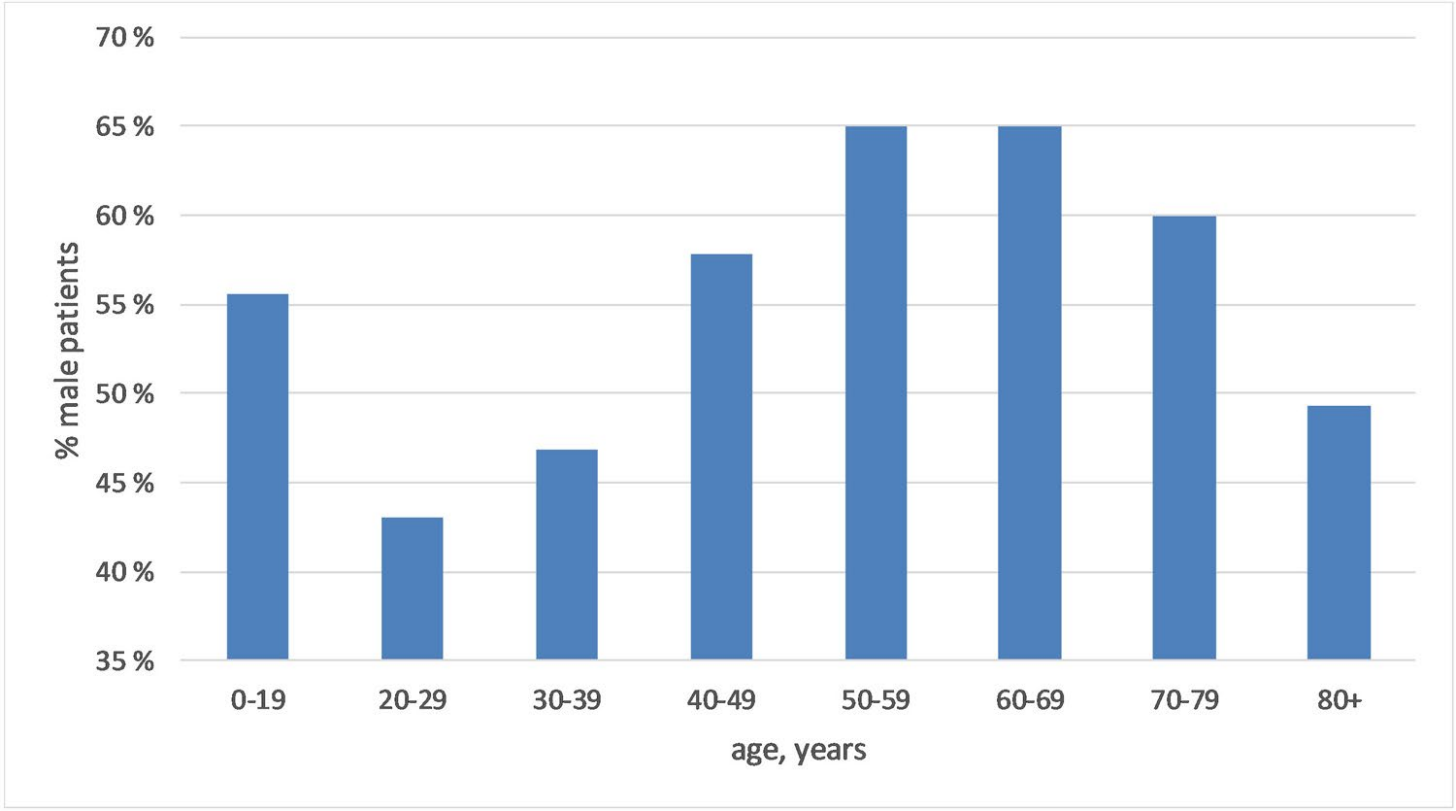
Sex distribution of eyes of patients with RRD in various age groups. For the age group 40–49, the sex distribution was significantly different from equal with *p* = 0.01, for the age groups 50–59, 60–69, and 70–79 with *p* < 0.0001. The other age groups were not significantly different from equal distribution. (Chi-square tests for one-dimensional tables)

Overall, 3360 of 5502 eyes (61.1%) with RRD occurred in male patients. The proportion of male patients with RRD in various age groups is shown in Fig. 3. In the age group with RRD below 20 years, the proportion of boys was 56%. Between 20 and 39 years, 55% of the patients were female. Between 50 and 79 years, a majority of 64% was male. In the oldest group over 80 years, no gender predominance was observed (49.3% male).

### Effect of age and sex on success rates

**Fig. 4:**
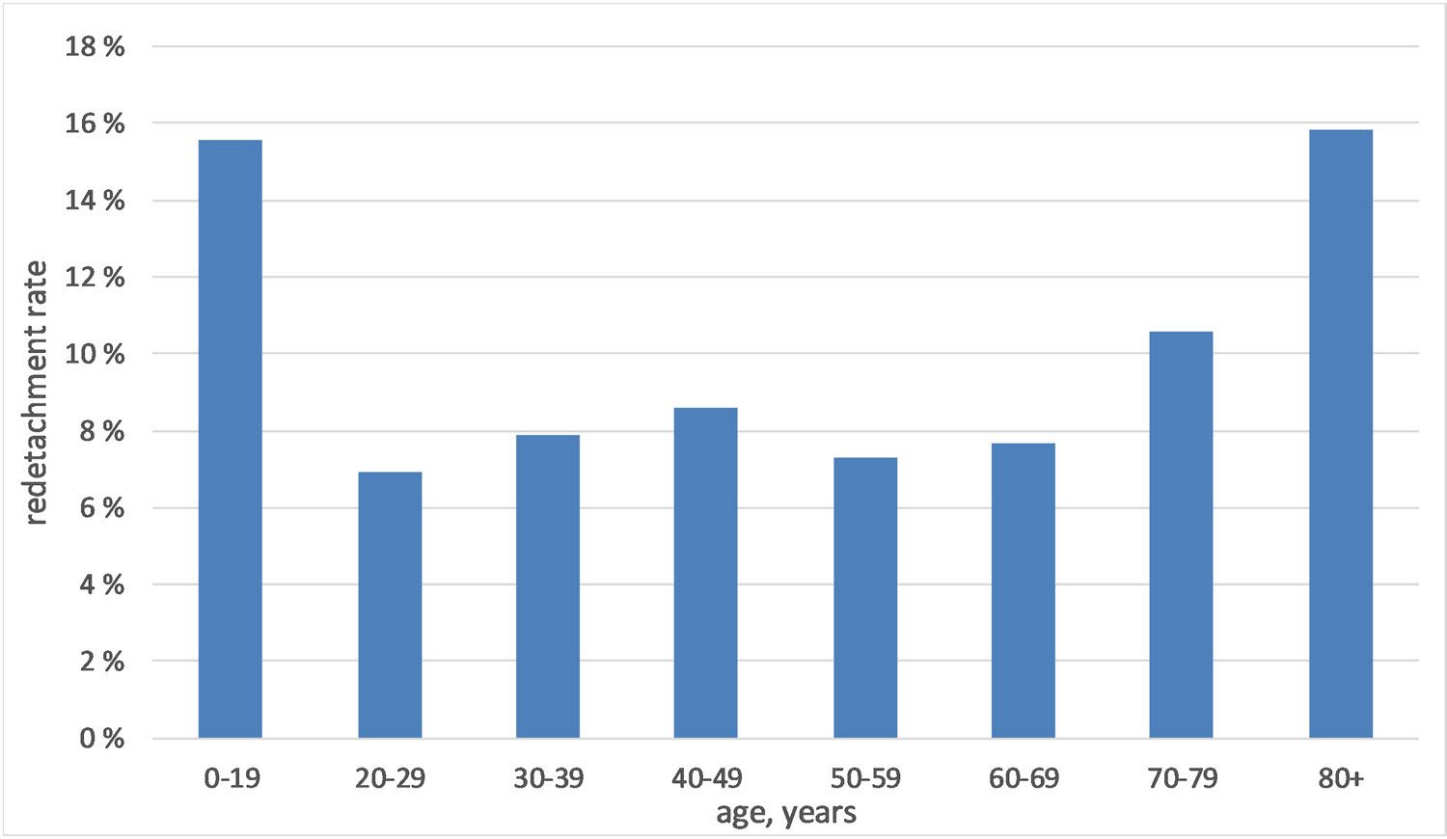
Redetachment rates of eyes of patients in various age groups

Overall, primary success rate after one surgery was 91.2% (5018 of 5502). Figure 4 shows the rate of redetachments in different age groups. The primary success rate was lower for the youngest (age 0–19: success rate 84.4%; 38 out of 45) and oldest patients (age 80 + : success rate 84.1%; 292 out of 347) compared to the middle-aged group (age 20–79: success rate 91.7%; 4688 out of 5110). Primary failure rate of RDD surgery was 305/3360 (9.1%) in male patients and 179/2142 (8.4%) in female patients, which was not statistically significantly different (*p* = 0.4, Pearson chi-square test).

### Lens status and sex

**Fig. 5:**
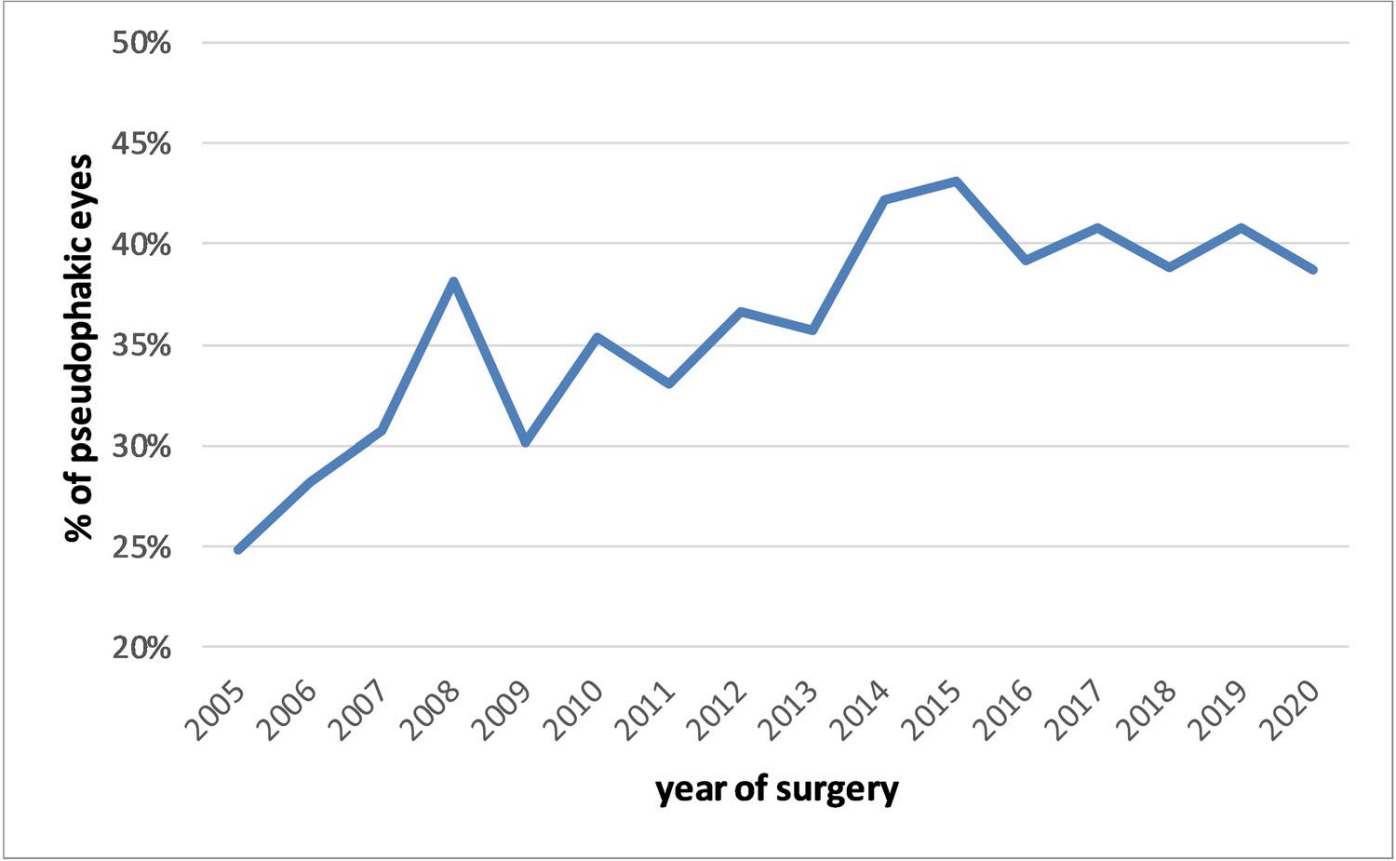
Proportion of pseudophakic lens status in eyes with RRD between 2005 and 2020

3398 (61.7%) eyes were phakic, 27 (0.5%) aphakic, and 2077 (37.7%) had an artificial intraocular lens. The percentage of pseudophakic RRD has increased over the observation period from 25 to about 40%. The increase in pseudophakic RRD occurred mainly between 2005 and 2014. Thereafter, it remained relatively stable (Fig. 5).

Of the pseudophakic RRDs, 67.3% occurred in male patients, while the proportion of male patients was only 57.3% in the phakic RRDs. Since the sex ratio varied between age groups (see Fig. 3), we separately analyzed the age group 50–69 years of age. In this subset, the pseudophakic detachments were male in 786 out of 1079 cases (72.9%), while from the phakic eyes in the same age group, 1285 out of 2110 (60.9%) were male.

### Lens status and success rates

Patients with pseudophakic RRD repair were on average 67 ± 11 (mean ± SD, range) years old. The phakic patients who had combined surgery were 63 ± 9 years old. Patients without cataract surgery during RRD repair were 49 ± 13 years of age. Mean age of the patients and redetachment rates for various subgroups are shown in Table 1. Notably, the primary success rate was higher in the phakic eyes that underwent phacovitrectomy (*n* = 2163; 93.0%), compared to those who were left phakic during vitrectomy (*n* = 451; 88.7%). This difference was statistically significant (*p* = 0.002; Pearson chi-square test).

**Table 1:** Age and redetachment rates of various subgroups depending on lens status and surgical technique

	Number of eyes	Redetachment rate, %	Age, mean ± SD
Treated with buckle surgery	810	10.9	49.2 ± 16.5
Pseudophakic, treated with vitrectomy	2051	9.2	66.7 ± 11.4
Aphakic, treated with vitrectomy	27	18.5	49.2 ± 15.2
Phakic, treated with vitrectomy, no lens surgery	451	11.3	49.4 ± 12.9
Phakic, treated with combined phacovitrectomy	2163	7.0	62.8 ± 9.4

## Discussion

In this study, we provide data from a retrospective analysis of over 5000 consecutive cases who underwent primary RRD repair at a single university hospital. Mean age (61 years) and male predominance (proportion of 61%) is in agreement with other series in the literature [2]. However, a more differentiated analysis shows that there is no homogeneous sex distribution over the whole age spectrum.

For children, retinal detachment is found more often in boys [5]. RRD in small children is commonly associated with vitreoretinal degeneration/dysplasia. Since in our series complicated RD cases were excluded, and some of them (trauma, Coats disease) are more commonly found in boys, the male predominance in children was less pronounced compared to other publications [5].

For young adults, most cases of RRD are due to atrophic round holes with an attached vitreous. This type of RRD is usually slowly evolving in myopic patients. Ung et al. showed that the mean age lies between 25 and 35 years and scleral buckling procedures remained highly effective in this selected group of patient [6]. Interestingly, this type of RRD was found predominantly in females [6–8]. In the present study, we also found a trend (not statistically significant) of female predominance (55%) in the age group 20–39 years of age.

The majority of RRD occurs in the age group over 50 in association with a PVD and traction-induced retinal tears. Between 50 and 79 years of age, 64% of the RRD patients in our study were males. In the age group over 80, we found both sexes were equally common affected, although the proportion of males is only 38% in the general population in Germany according to the “Federal Office for Stastistics.” Thus, if the sex distribution in the population in this age group is taken into account, men over 80 years of age also have a higher individual risk for RRD than women (*p* = 0.003, chi-square test).

RRD has been consistently shown to be more common in male patients. This fact persisted after exclusion of pseudophakia and trauma. It has been speculated that males had a longer axial length and this could explain the male predominance in RRD [9]. However, this would also be a risk factor for atrophic round hole RRD with attached posterior vitreous in young adults, but here females are at higher risk. Males are more affected by PVD associated RRD with tractional tears. Anatomical changes with a more posteriorly located vitreous base in males have also been described [10] and would provide a more convincing explanatory approach.

The number of cataract surgeries has increased in the order of 10% per year over the last decades in industrialized countries [11]. Increasingly, eyes with less advanced lens opacities and better vision are operated. In particular, eyes with high refractive errors undergo more commonly clear lens extraction, with highly myopic eyes being at highest risk for RRD. Since cataract surgery is a major risk factor for RRD, it is inevitably that the proportion of pseudophakic RRD increases with time [1]. Here, we show an increase from 25 to 40% pseudophakia in RRD eyes over the observation period.

Notably, in the age group 50–69, a proportion of 73% of the patients with pseudophakic detachments was male, while in phakic eyes in the same age group, only 61% were male, although the majority (about 60%) of all cataract surgeries is performed in females in Europe [12] and the USA [13]. A similar phenomenon was observed in Scotland, where within the pseudophakic group, the overrepresentation of men was even more marked (M:F, 2.3:1), despite a higher rate of cataract surgery in women in the UK [14]. Although the present study was not designed to evaluate this question, a possible explanation for the data could be that males in this age group have a higher risk for RRD than females, which is further increased by cataract surgery. This effect appears less pronounced for women. Cataract surgery is believed to increase the RRD risk by accelerating vitreous degradation and PVD. Therefore, it would be plausible that sex differences in the vitreous base anatomy [10] might play a role for the increased RRD risk for men.

In our study, the overall primary anatomic success rate was 91%, which is in accordance with other recently published studies for RRD surgery [2]. The high proportion of vitreous surgery follows a general trend from buckle to vitrectomy [3]. No difference was observed in success rates of RD surgery between male and female patients.

The redetachment rate in the very young and the very old age group was somewhat higher. For children, lower success rates have been previously observed [5]. This may be due to more atypical findings and a delayed diagnosis in children. In the very old, there is probably a higher rate of ocular and general comorbidities, leading to later diagnosis and more difficult ocular situations (e.g., PEX syndrome, cloudy cornea, and narrow pupil). A reduced capacity of the RPE to pump out subretinal fluid and reattach the retina may also play a role. A recent analysis of BEAVRS data also showed lower anatomical success rates in the age group over 80 with RRD [2].

The success rates also showed some correlation with the lens status. In aphakic eyes, success rate was lower. In the present series, aphakic eyes were not generally considered to have complicated RRD and therefore were not excluded. They are only a small group of eyes, but may probably present with more confounding factors due to previous anterior segment trauma, narrow pupil, aphakia due to Marfan’s syndrome, or a congenital cataract. This may explain the higher redetachment rate. The small differences between buckle surgery, and vitrectomy in phakic and pseudophakic eyes are probably not relevant, considering that the groups differ in their initial situations.

A controversial question is whether cataract surgery should be performed simultaneously during vitreous surgery for RD. Vitrectomy leads to accelerated cataract formation. Although not fully proven, it is assumed that increased oxygen diffusion to the lens may play a role [15]. Cataract formation after vitrectomy appears to be age dependent, with older patients developing lens opacifications earlier, often within a few months. Less expected, after buckle surgery also a high frequency of cataract surgery was observed [16]. Therefore, especially in patients beyond the age of accommodation, RRD may be operated primarily combined as phacovitrectomy. This saves the patient an additional surgery, is more cost effective, and reduces time of reduced vision [17]. Especially in times of pandemic, it can reduce the risk for nosocomial infection by avoiding additional hospital visits [18].

A major intraoperative advantage of combined surgery is uninhibited view and access to the most peripheral retina and the vitreous base which allows a nearly complete removal of vitreous traction and identification of retinal tears. In a pseudophakic eye, the peripheral secondary cataract will obscure the view, and in phakic eyes, the complete release of traction to the vitreous base is hardly possible without risking to touch the lens. This may lead to better primary reattachment rates of phacovitrectomy. On the other hand, combined surgery requires broader surgical skills and has a higher potential to create intraocular inflammation which in turn may theoretically increase the risk for PVR development.

Cataract surgery months or years after vitrectomy, when the lens is opaque, may be more challenging due to missing vitreous support of the lens and hard lens nucleus, both leading to increased complications in cataract surgery. On the other hand, disadvantages of phacovitrectomy may be a reduced red light reflex due to vitreous hemorrhage or bullous detachment of the retina. Postoperative synechiae or anterior dislocation of the IOL due to gas pushing forward with iris capture may occur.

In addition, calculation of the IOL may be more difficult, especially if the macula is detached. Here the fellow eye may be used for measuring axial length. The number of eyes having undergone refractive surgery is increasing which also could complicate IOL calculation. Since RRD often occurs in highly myopic eyes, the selection of the intended refraction after lens surgery is difficult. An intended emmetropia or low myopia will induce anisometropia, which necessitates contact lens correction or subsequent refractive lens surgery in the healthy fellow eye, which in turn will expose this eye to an even higher risk for RRD. Explaining this complex situation to a nervous patient in an emergency situation can be challenging.

The literature does not consistently recommend to perform cataract and vitreous surgery in one or two steps. Some studies report a lower success rate of combined surgery [19, 20]. In addition, combined surgery had less favorable refractive outcome [20, 21] and there was a higher complication rate for inexperienced surgeons [22]. In a scenario-related survey of BEAVRS, most vitreoretinal surgeons would avoid combined surgery in RDD cases with a history of laser-assisted refractive surgery, even when faced with a visually significant cataract [23]. Other authors had better results with phacovitrectomy compared to vitrectomy alone [24]. Most studies however, including a small randomized trial [25] and a recent meta-analysis [26], found no difference in redetachment rates between the two approaches [22, 27].

In the present study, we changed strategy in 2008 to 2010 and performed phacovitrectomy in most eyes over 50 years of age, even if the lens still was clear. When comparing the redetachment rates, we found better results in the group operated with combined surgery (7% vs 11% redetachment rate). One has to keep in mind however that the groups were not randomized and differed in several aspects. The phacovitrectomy patients were generally older and operated later in the observation period and the absolute differences were small.

Limitations of our study are its retrospective design, the lack of a stringent follow-up of the patients, the missing information of preoperative PVD status, and missing information about visual acuity and functional outcome. However, we present a large number of RRD cases over a 15 years period from a single center and have focussed on data that can be obtained reliably in retrospective from the patients’ charts.

In conclusion, our analysis of the influence of lens status, age, and sex in a large consecutive series of RD surgery revealed that the gender predominance in RD varies within different age groups. While in children, more boys were affected; in young adults, there were more females with RD; and in patients over 50 years of age, a strong male predominance was observed. The male predominance in RRD is stronger in pseudophakic than in phakic eyes. The proportion of pseudophakic RD has increased during the observation period from 25 to 40%. In phakic eyes with RRD combined phacovitrectomy yielded better anatomical results than vitectomy alone.
